# Neuroprotection in neurodegenerations of the brain and eye: Lessons from the past and directions for the future

**DOI:** 10.3389/fneur.2022.964197

**Published:** 2022-08-12

**Authors:** Leonard A. Levin, Christopher Patrick, Nozhat B. Choudry, Najam A. Sharif, Jeffrey L. Goldberg

**Affiliations:** ^1^Departments of Ophthalmology and Visual Sciences, Neurology & Neurosurgery, McGill University, Montreal, QC, Canada; ^2^TELUS Health, Ottawa, ON, Canada; ^3^Global Alliances and External Research, Ophthalmology Innovation Center, Santen Inc., Emeryville, CA, United States; ^4^Spencer Center for Vision Research, Byers Eye Institute, Stanford University, Palo Alto, CA, United States

**Keywords:** neuroprotection, neurodegeneration, translational failure, Alzheimer's disease, Parkinson's disease, amyotrophic lateral sclerosis, optic nerve, retina

## Abstract

**Background:**

Neurological and ophthalmological neurodegenerative diseases in large part share underlying biology and pathophysiology. Despite extensive preclinical research on neuroprotection that in many cases bridges and unifies both fields, only a handful of neuroprotective therapies have succeeded clinically in either.

**Main body:**

Understanding the commonalities among brain and neuroretinal neurodegenerations can help develop innovative ways to improve translational success in neuroprotection research and emerging therapies. To do this, analysis of why translational research in neuroprotection fails necessitates addressing roadblocks at basic research and clinical trial levels. These include optimizing translational approaches with respect to biomarkers, therapeutic targets, treatments, animal models, and regulatory pathways.

**Conclusion:**

The common features of neurological and ophthalmological neurodegenerations are useful for outlining a path forward that should increase the likelihood of translational success in neuroprotective therapies.

## Background

Neuroprotective strategies involve the use of a broad range of therapeutics to promote neuronal survival through the preservation and restoration of neuronal structure and function (1, 2). Preservation involves the maintenance of current neuronal function, either by promoting or enhancing neuronal survival (3). For optic neuropathies, neuroprotective therapies could be directed at improving visual function where there has been a decrement due to past damage from disease by increasing function using existing neuronal wiring, thereby increasing activity for visual perception (4). Neurorestoration involves replacement of components involved in neuronal circuitry to repair the damage or activate or regenerate residual function (3). Neuroregenerative strategies for optic neuropathies include enabling retinal ganglion cells (RGCs) to regenerate and form new synapses with the brain, or the formation of new RGCs from either extrinsically or intrinsically provided stem cells (4, 5).

Neuronal diseases are difficult to cure, in part due to disease complexity and the multiplicity of prevailing pathological insults on the neuronal elements at the structural and functional levels (1, 6–8). Neurodegenerative diseases involve injuries or insults that affect multifactorial biochemical processes, critical signal transduction cascades, and complex interactive associations within the neurons and their axons, dendrites and synapses, glial and immune cells, and blood vessels that constitute the central nervous system (CNS) (1, 9). To date, no therapies that are directly neuroprotective have been approved by regulatory agencies, as none are capable of fully restoring neuronal function; most available treatment options are symptomatic, with even approved drugs for neurodegenerations having limited clinical impact on the progression of neuronal diseases (1, 4).

There are common challenges faced by neurology and ophthalmology, including understanding and treating neurodegenerative diseases, as well as translating laboratory work to the clinical arena (10). There is a clear need to extract insights from past failures relating to neuroprotection in those fields to help improve the translational potential of emerging therapies (7, 10, 11). In response to this need, we organized a meeting that would bring together experts on neuroprotection from both the neurological and ophthalmological spheres. This meeting was held virtually on September 1-2, 2020 and was followed by a series of discussions to develop a manuscript that would be useful to the neuroprotection community. This review summarizes the key topics from the meeting and outlines several considerations for improving neuroprotective therapy development and thereby advance the treatment of neurodegenerative diseases.

## Main text

### Commonalities between ophthalmology and neurology and lessons from past failures

#### Commonalities between neurodegenerations in ophthalmology and neurology

Chronic neurodegenerative diseases are often characterized by the loss or dysfunction of particular subsets of neurons (12). The most common neurodegenerative diseases span structures and neuronal subgroups from eye to brain, including in approximate order of prevalence, Alzheimer's disease (AD), glaucoma, age-related and inherited macular degenerations, Parkinson's disease (PD), and others. For the visual system, degeneration often involves neuronal loss in the retina and optic nerve, and secondarily in higher visual centers of the brain; progressive stages of neuronal dysfunction and death lead to eventual blindness in late stages when changes become irreversible (13). In both cases, clinical characteristics and patient population features are often shared, such as a predilection for many neurodegenerations to increase in incidence with increasing age, and to have chronic courses measured in years to decades.

The pathophysiology of neurodegenerative diseases in the eye and brain clearly differ, but here again shared biological themes have emerged. For example, vascular insufficiency, failure of mitochondrial function, loss of axon transport, and degenerative retraction of dendrites and/or axons have all been variously implicated in both preclinical models and human clinical or postmortem measures of retinal, optic nerve, and brain-centered diseases. In some cases, direct cellular links have been identified, such as mild atrophy of retinal ganglion cells in AD, and mild cognitive impairment in glaucoma patients (14). Molecular/genetic links similarly point toward shared underlying pathophysiology, such as mutations in some mitochondrial fission/fusion proteins leading to dominant optic atrophy, and in others leading to PD.

Similarly, preclinical support for candidate neuroprotective therapies are often shared between diseases of the eye and brain. Various neurotrophic factors, antioxidants, apoptosis inhibitors, and kinase and other signaling enzyme inhibitors, among many other classes of therapeutics, have shown similar strong efficacy across neurodegenerative diseases (13, 15, 16). These data further support the hypothesis that downstream pathophysiology is shared across diseases independent of upstream or inciting insults. Thus, the premise of looking across the aisle between eye and brain neurodegenerative diseases for basic and translational insight is strongly supported.

#### Neuroprotection trial failures despite rational and/or supported therapies

Even well-designed clinical trials and rational therapies can fail for a variety of reasons (1, 10). Over the past few decades, numerous neuroprotective pharmacologic agents have been investigated in the laboratory in animal models of human disease and in human clinical trials for acute (e.g., stroke, head, and spinal cord injury) and chronic (e.g., AD and PD) neurologic diseases, as well as retinal and optic nerve diseases (e.g., diabetic retinopathy, glaucoma, and other optic neuropathies) (11, 17). Though many agents demonstrated early promise in limiting neuronal damage in animal models, promising preclinical results have almost invariably failed to translate to the clinic (4, 6, 7, 11). The historic failures of neuroprotection across different diseases suggest potential flaws in the present approach to neuroprotection and structural barriers that must be overcome (10). Many conceptual and methodological challenges have likely contributed to the difficulties in translating animal model-based experimental results to the clinic (9). Lessons from past clinical trial experiences afford an opportunity to integrate acquired knowledge into the planning of future neuroprotection trials for neurological and ophthalmic diseases.

#### Lessons from neuroprotection clinical trials in ophthalmology that demonstrated no efficacy

##### Diabetic retinopathy

Two randomized, double-masked, placebo-controlled phase-3 trials investigated the effect of ruboxistaurin, a protein kinase C beta inhibitor, on slowing the development of diabetic macular edema, a complication of diabetic retinopathy, and its attendant visual loss (18). In the combined studies (*N* = 1,028), sustained moderate visual loss occurred in 4.4% of placebo- vs. 2.3% of ruboxistaurin-treated patients (*P* = 0.069) (18). The magnitude of effect of ruboxistaurin on vision loss was consistent with what was observed in two prior studies (approximately 50% reduction beyond standard care). However, event rates were low and statistical significance was not achieved (18). A challenge *post-hoc* is differentiating between anatomic success, e.g., prevention of edema, and functional success, e.g., slowing vision loss.

##### Leber hereditary optic neuropathy

An open-labeled, non-randomized prospective pilot study investigated the use of topical brimonidine purite, an α-2 adrenoreceptor agonist, as a prophylaxis for second eye involvement in LHON, a maternally inherited, blinding, bilateral optic neuropathy (19). Treatment with brimonidine (0.15%) 4 times daily in the unaffected eye for up to 2 years was unsuccessful in preventing second eye involvement in recently monocularly-symptomatic LHON (19).

RESCUE was a multicenter, randomized, double-masked, sham-controlled, phase-3 clinical trial investigating the efficacy of a single intravitreal injection of rAAV2/2-ND4 (a gene therapy vector enabling allotopic expression and delivery of the wild-type ND4 protein to mitochondria within RGCs) in subjects with visual loss from LHON (20). At 96 weeks after unilateral injection of rAAV2/2-ND4, LHON subjects carrying the m.11778G > A mitochondrial DNA mutation treated within 6 months after vision loss achieved comparable visual outcomes in the injected and un-injected eyes (20). The primary endpoint of a −0.3 logMAR difference between rAAV2/2-ND4–treated and sham-treated eyes was not met (20). These trials were well-designed and enrolled appropriate patient participants, suggesting that the lack of sufficient efficacy was unrelated to structural trial issues.

##### Glaucoma

Two randomized, double-masked, placebo-controlled, parallel-group, multicenter, 48-month studies were identically designed, initiated 1 year apart, and completed in 2006 (21). Their purpose was to evaluate the efficacy and safety of oral memantine, an N-methyl-D-aspartate receptor-channel inhibitor, as a potential neuroprotective agent in open-angle glaucoma (OAG) at risk for progression (21). These phase 3 trials, which lasted more than 5 years, did not reveal a significant benefit for memantine treatment in preventing the progression of visual field loss in patients with glaucoma (22). Overall, progression of glaucomatous findings assessed by standard automated perimetry (SAP), frequency doubling technology, or stereoscopic optic disc photographs did not reveal a consistent protective effect of memantine, and the primary endpoint was not met in either study or in a pooled analysis (21). Failures such as these have since raised awareness around issues of trial design, patient inclusion, and strength and consistency of preclinical data (23).

##### Non arteritic anterior ischemic optic neuropathy

Clinical trials investigating the neuroprotective role of brimonidine have failed to translate the efficacy observed in animal models into similar efficacy in humans (24). A 3-month, double-masked, placebo-controlled, randomized European multicenter trial investigated the efficacy and tolerability of brimonidine tartrate (0.2%) for the treatment of NAION (25). A statistically significant benefit for visual acuity in patients receiving brimonidine tartrate was not observed (25). While better visual field results were observed in the brimonidine group, these were non-significant trends, both statistically and biologically (25).

##### Example of a neuroprotection clinical trial in ophthalmology that demonstrated some success but was flawed

A randomized, double-masked, multicenter clinical trial compared the effects of alpha2-adrenergic agonist brimonidine tartrate (0.2%) with those of the non-specific beta-adrenergic antagonist timolol maleate (0.5%) in preserving visual function in patients with low-pressure (“normotensive”) glaucoma (26). Statistically fewer brimonidine-treated patients (*n* = 9, 9.1%) had visual field progression than timolol-treated patients (*n* = 31, 39.2%, log-rank 12.4, *P* = 0.001) (26). However, more brimonidine-treated (*n* = 28, 28.3%) than timolol-treated (*n* = 9, 11.4%) patients discontinued study participation because of drug-related adverse events (*P* = 0.008) (26). While baseline characteristics were similar between dropout patients in both treatment groups, failure to obtain information from this subset of patients limited interpretation of the results (26). Thus, despite demonstrating some effect with brimonidine treatment, these and other caveats led to it not being widely accepted or translated into clinical practice (27).

#### Lessons from neuroprotection clinical trials in neurology that demonstrated no efficacy

With the exceptions of memantine and various cholinesterase inhibitors (e.g., donepezil, galantamine, and rivastigmine) in AD, as well as riluzole (a benzothiazole derivative with neuroprotective and potential antidepressant and anxiolytic activities) and edaravone (a potent free radical scavenger/antioxidant) in amyotrophic lateral sclerosis (ALS), clinical trials in neuroprotection have failed to demonstrate improved outcomes (28, 29). Recently, aducanumab was approved for AD, although the weak evidence supporting its efficacy has led to much controversy (30). Many agents in different stages/phases are being considered as potential disease-modifying agents in AD and PD, with some compounds having advanced to phase-3 trials (31, 32). There is a need for an effective treatment, and large investments in basic research and drug development have been made (33). However, there has been limited progress from a clinical perspective, marked by the clinical efficacy failure of several late-stage drug candidates, ultimately resulting in no new therapies being approved by the US Food and Drug Administration (FDA) in over a decade (33).

##### Alzheimer's disease

DIAN-TU-001 was a phase-2/3 study that compared investigational therapies (gantenerumab and solanezumab; human immunoglobulin-1 antibodies directed at sequestering amyloid-β fibrils) with placebo to determine if either treatment could slow the rate of cognitive decline and improve disease-related biomarkers in patients with a genetic mutation for inherited AD (30). The study did not meet its primary endpoint in patients with an early-onset, inherited form of AD and did not show a significant slowing of the rate of cognitive decline in patients treated with gantenerumab compared with placebo (30).

A double-blind, randomized, multicenter, clinical trial with 24 months of treatment and follow-up investigated the efficacy of minocycline (a tetracycline antibiotic) treatment in modifying cognitive and functional decline in patients with mild AD (34). Minocycline treatment did not significantly delay progression of functional and cognitive impairment compared with placebo (34).

AMARANTH and DAYBREAK-ALZ were randomized, placebo-controlled, phase-2/3 and phase-3 trials investigating the potential of lanabecestat (an oral β-secretase cleaving enzyme inhibitor) to slow the progression of AD compared with placebo in patients with early AD (mild cognitive impairment) and mild AD dementia (35). Treatment with lanabecestat was well tolerated but did not slow cognitive or functional decline (35). Together these trials illuminated challenges in translation, including trial design, biomarker, and preclinical data translatability.

##### Multiple sclerosis

A phase-2b, multi-arm, parallel group, double-blind, randomized placebo-controlled trial conducted at 13 clinical neuroscience sites in the UK investigated the efficacy of 3 neuroprotective agents [amiloride 5 mg (a diuretic), fluoxetine 20 mg (a serotonin-uptake inhibitor), riluzole 50 mg (a tetrodotoxin-sensitive Na^+^-channel blocker with anti-anxiety and antidepressant activity)] in patients aged 25–65 years with secondary progressive multiple sclerosis (36). No difference was observed between any active treatment and placebo, and the authors suggested that the absence of evidence for neuroprotection in this adequately powered trial indicated that exclusively targeting these aspects of axonal pathobiology in patients with secondary progressive multiple sclerosis was insufficient to mitigate neuroaxonal loss (36). Whether targeting multiple pathways with a combination therapy may work more effectively remains to be tested.

##### Parkinson's disease

A multicenter, randomized, parallel-group, double-blind, placebo-controlled trial investigated the effect of isradipine, a dihydropyridine calcium-channel blocker, on the rate of clinical progression of PD (37). Long-term treatment with immediate-release isradipine did not slow the clinical progression of early-stage PD in previously untreated patients (37).

##### Amyotrophic lateral sclerosis

EMPOWER was a randomized, double-blind, placebo-controlled phase-3 trial that investigated the efficacy and safety of dexpramipexole (R-enantiomer of a dopamine receptor agonist) in patients with familial or sporadic disease (with ALS onset 24 months or less before baseline) (38). Although dexpramipexole was generally well tolerated, its effects did not differ from placebo on any prespecified efficacy endpoint measurement (38).

A double-blind, randomized, placebo-controlled, multicenter trial of a duration of 18 months investigated the efficacy and safety of olesoxime (a cholesterol derivative used for the preservation of mitochondrial function) in patients with ALS treated with riluzole (39). Survival was not significantly different between treatment arms (*P* = 0.71); estimated overall survival was 67.5% (95% CI: 61.0–73.1%) in the placebo group and 69.4% (95% CI: 63.0–74.9%) in the olesoxime group (39).

### Understanding roadblocks at the basic research level and clinical trial level

There are different reasons why candidate neurotherapeutics fail to translate in clinical trials to demonstrate benefits for patients. Despite significant investments in basic scientific research, technological advances, and overall enhanced knowledge of human diseases, translation of these findings into therapeutic advances has been slower than expected, and the return on this investment has been limited with regard to clinical impact (40). This failure to translate can be attributed to a number of roadblocks common between ophthalmology and neurology trials.

#### Type 1 and type 2 translation problems

There are two types of translation problems: type 1 and type 2 (41). Type 1 translational problems occur when there are problems with either the preclinical data (bench) or the clinical trial data (bedside) (41). This type of problem is suggestive that preclinical or clinical trial data were unconvincing due to flaws in experimental design or data interpretation (41).

In the case of memantine in glaucoma, in retrospect, the preclinical data were not completely convincing in non-human primates; the positive treatment effects observed at very high intraocular pressure (IOP) could have been representative of neuroprotection against ischemia, which is consistent with the mechanism of action of such therapies in preclinical models of ischemia (42, 43).

In the case of brimonidine in NAION, the human clinical trial results were inconclusive, and a statistically significant benefit in patients receiving brimonidine was not demonstrated. After unmasking the data, the investigators chose to halt the trial due to poor recruitment (24, 25).

With type 2 translational problems, the preclinical data and clinical data are individually satisfactory, but the findings are incongruent with each other, and there are problems when shifting the results from bench to bedside (41). This type of problem is suggestive that there are discrepancies in results between different animal models and difficulties when applying animal findings to humans (41). In some ways this should be expected. Animal models of disease often only use a single insult (e.g., chemical, mechanical, or pathological), against which the potential protective agent is tested. In reality, human neurological or ophthalmic disorders are etiologically manifested due to multiple ongoing insults occurring at multiple neuroaxonal sites in the retina and brain nuclei (1, 6–8).

An example of a type 2 translation problem involving a lack of efficacy at the phase-3 clinical trial stage was seen with dexpramipexole and olesoxime. These were agents that demonstrated mitochondrial function-dependent neuroprotective activity in the SOD1^G93A^ mouse model of ALS, which served as a rationale for initiating clinical development (44). However, these compounds failed to demonstrate a clinical benefit in patients with ALS (44).

An example of a type 2 translation problem involving translational differences between preclinical and human models in drug delivery was seen with isradipine. Here, the dose used in the STEADY-PD III study could have been insufficient to engage the target calcium channels associated with neuroprotective effects in preclinical settings (37). A method for directly measuring target calcium channel engagement with systemic isradipine administration in humans does not exist, raising the possibility that its brain bioavailability was lower in humans than in preclinical models (37).

#### Potential reasons for neuroprotection trial failures

Although many trials fail because of underpowering, choice of an inappropriate outcome measure(s), patient selection issues, and many other familiar trial design issues, there are several other less familiar reasons for translational failure that are relevant to neuroprotection in neurodegenerations.

##### Diagnosis and trial entry delays

The diagnosis of neurodegenerative diseases is often delayed and is generally reported years or even decades after commencement of neuronal destruction. By the time patients enter a clinical trial, their disease may be too advanced for a therapy to be effective (45). Neuroprotective therapies should be initially administered at the earliest stage of disease when they will be most effective in delaying progression (1, 13). However, patients are not typically seen in the clinic until mid- to late-stages of disease, when the chances of significantly halting neurodegeneration are minimal (1, 13).

##### Limitations of testing methods and shortcomings of animal models

A major challenge in translating neuroprotective strategies to the clinic is the need for sensitive tools, e.g., for retinal screening and disease monitoring (17). The quality of research findings is predominantly contingent on the quality of the input data and the methods for their processing and interpretation (40). To determine whether an agent is neuroprotective, it is imperative to have reliable methods to detect its efficacy (46). The improper use of statistical analysis methods and the misinterpretation or misuse of *p*-values can lead to inaccurate conclusions, thus adding to reproducibility challenges (40).

While mouse models have some value in helping researchers understand disease pathobiology and the mechanism of action of therapeutic candidates, they have mostly failed as a translational tool, and their predictive utility has been suboptimal (40). Even with a useful animal model, failures can occur due to an incomplete understanding of disease pathophysiology. Moreover, no available models mimic the full spectrum of neurodegenerative pathology (6–8, 46). There is no perfect model for any of the neurodegenerative diseases, as each one fails to capture the full spectrum of pathological components involved (6, 7, 11).

##### Inherent progressive variability associated with translational science

The methods used along the trajectory from preclinical to clinical research are associated with progressively larger amounts of variability (47). Differences in scale, timing, and choice of model can make it difficult to confidently extrapolate findings from animals to humans (47). Human research participants not only differ genetically and epigenetically, but also display heterogeneity in terms of how long they take to become symptomatic and present for treatment, how sensitive or susceptible to damage their neuroaxonal components are to the burgeoning insults they have to endure during disease initiation and progression, how well they comply with therapy, the degree of placebo effect, and other factors (6–8, 47).

##### The nature of the translational process

Promising therapeutic strategies in recent years have failed to translate into effective treatments, and while studies may be well-intentioned, there are subtleties that may be missed when performing experiments or interpreting which can be consequential (40, 48). Methodological flaws and poor experimental design in preclinical studies can lead to systematic bias, thus leading to irreproducible and unreliable data as well as inaccurate conclusions (40). The culture of preclinical research is markedly different from that of clinical research (e.g., differences in the training of the participants, how hypotheses are generated and tested, and the emphasis on repeatability). These differences between cultures are reflected in how experiments are designed and results interpreted (47, 49–51). The lack of target relevance or disease mechanism can lead to high heterogeneity within a patient population (40). In addition, the lack of publications of many negative clinical trials can lead to inadvertent repetition of the same study rationale, thereby wasting valuable time and resources (45).

##### Complexities of human disease

Diseases are multifactorial, involve multiple insults, and occur simultaneously in RGCs and/or axons. However, as noted above, experiments are typically performed using a model involving a single insult (6–8). In the case of neurological diseases such as ALS, where there are challenges related to complex genetic associations, patients with variable clinical subtypes are often pooled in the same study group, which can lead to significant statistical differences in the observations (45). Furthermore, neurodegenerative diseases progress very slowly, over many months and years (1, 2). Thus, neuroprotective studies must follow subjects for a sufficient length of time, so that measurable differences in outcomes can be ascertained between treatment groups. For most neurodegenerative diseases, without selecting for a “fast progressor” subset, this may require more than 5 years, which is longer than typical grant/funding cycles (41). Producing longitudinal data in diseases with extended lifetime risks requires decades, but funding agencies or pharmaceutical companies rarely operate on such timelines.

##### Complexities of the central nervous system and visual system

There are numerous specialized neuronal cell types and subtypes that give rise to a complex connectome and thereby a complex cognitive, sensory, or motor function. Due to the heterogeneity and complexity of mammalian neural cells, neuronal and glial classification has been challenging, and many subtypes have not yet been characterized (52). Despite superficial similarities between animal and human visual systems, there is divergence in terms of anatomy, physiology, genetics, and disease manifestation (53). As with neurons in the brain, RGCs are also highly diverse, and there is a knowledge gap in understanding which cell types are most relevant for preservation of visual capacity and whether different cell types display distinct susceptibilities to disease or trauma (52). For example, effective neuroprotection of RGCs will potentially require targeting of multiple dysfunctions contributing to neurodegeneration (54). For RGC replacement, the challenge has been converting stem cells to RGCs and establishing both structural and functional connections to the optic nerve and brain thalamic nuclei and ultimately the visual cortex (3, 53, 55).

##### Acceptance of biomarkers and endpoints by regulatory bodies

In terms of glaucoma clinical trials, there is no example of a primary outcome measure for neuroprotection which has led to FDA approval. All approved drugs were based on IOP-lowering efficacy (54). However, this outcome is irrelevant for neuroprotection, for which an objective measure of visual loss, either functional and/or structural, is needed (54). The most studied structural biomarker for glaucoma is optical coherence tomography (OCT) of the retinal nerve fiber layer (RNFL). Despite many good prospective studies showing it correlates with visual fields, OCT of the RNFL is currently not recognized by the FDA as a primary outcome measure for glaucoma. Even with a good biomarker that is clearly related to the disease and whose variability is well-characterized, these surrogate biomarkers have not reached the requisite threshold. Thus, any other biomarker proposed would need to surpass that for a pivotal outcome (56). Furthermore, a therapy may have a biologically relevant impact in protecting a particular type of RGC, but may not have a clinical impact based on current FDA-approved endpoints.

There are many FDA-acceptable endpoints that are relevant in glaucoma (i.e., contrast sensitivity, patient-reported outcomes). However, there is insufficient data relating to some of these endpoints as compared to visual field in terms of testing reliability or measures of progression (56). In the case of glaucoma and many other diseases, there is probably no single “ideal” biomarker that will cover all aspects of clinical disease including early detection, severity prediction, progression, and response to treatment (57). The challenge with biomarkers, from a long-term perspective, is that what is needed are large-scale trials with gold-standard endpoints confirming that biomarkers correlate. These are expensive endeavors if trials are to be comprehensive (58).

#### Challenges in translating neuroprotection and neurorestoration

Across the fields of ophthalmology and neurology, innovative research is currently being conducted to advance our understanding of pathways and potential therapeutics in neuroprotection (4, 16, 31, 59). Despite recent progress, there are still many questions and challenges in terms of how to effectively approach neuroprotection and restoration of the visual system, both at the basic research level and clinical trial level.

##### At the basic science research level

To optimize research strategies, there is a need to differentiate between more robust neuronal cell subsets capable of enhanced survival and more vulnerable subsets that are more difficult to preserve (3, 60). While animal models have been useful in providing a greater understanding of disease pathology and drug mechanism of action, their predictive utility has fallen short of expectations (11, 40). Some questions will never be answered in rodents, but a better understanding could be gained using non-human primates. Non-human primates are probably best suited to mimic aspects of the human visual system because there are important anatomical similarities (51, 61, 62). However, questions remain as to whether the use of non-human primates in the study of neurodegenerative diseases could bring us closer to models that are more representative of human disease (61, 63, 64). Studies performed using monkeys have become prohibitive due to the costs of the animals and their husbandry, especially over a long-term basis needed to determine the neuroprotective efficacy of drug candidates (6–8).

There are challenges at the clinical trial as well. For example, for optic neuropathies there is a need to establish a clearer definition of effective neuroprotection (54). There are a number of questions that need to be discussed in optimizing clinical trial design (Table 1).

**Table 1:** Questions in optimizing clinical trial design.

Should we think about functional improvement rather than neuroprotection?
Is the treatment goal to simply restore sight or also restore higher levels of visual processing (e.g., the ability to recognize faces)?
What are the goalposts or benchmarks for bending the curve in the positive direction for the ophthalmology or neurology fields to get excited about a drug/therapeutic candidate?
Should we continue to seek a “master” overarching target or pathway to help launch an appropriate therapeutic discovery campaign, or should we continue to pursue a cocktail of numerous neuroprotectants as a treatment goal?
What should the target product profile look like for a neuroprotective therapeutic, and is that achievable?
What is an acceptable therapeutic index of a potential protective treatment modality?
Should we be agnostic about the route of delivery of the therapeutic agent(s), or is a particular route/site of delivery preferred?

### A path forward for greater success in developing effective therapeutics for neuroprotection

We are at a crossroads, and in order to advance, different approaches, clearly defined strategic plans, and actionable goals are needed to develop effective neuroprotective therapies (13, 65, 66). There are many areas of commonality between ophthalmology and neurology, and thus many potential avenues to collaborate and adopt new approaches to combatting common issues faced by each discipline in neuroprotection (10). There is a need to delineate a path forward toward developing effective therapeutics which can improve the lives of patients with neurodegenerative diseases. Next steps include developing recommendations, strategic plans, and actionable steps to which will lead to better biomarkers and better designs of neuroprotection trials (13, 67). The achievement of a consensus on how to design and execute translational research in neuroprotection could help optimize the allocation and use of resources, and accelerate the development and approval of effective neuroprotective agents (10, 56).

#### Therapeutic targets

Neuroprotective strategies generally explore therapies that stimulate cell survival pathways and rehabilitative methods that either increase endogenous repair mechanisms or inhibit cell death pathways, thereby enhancing the neuron's ability to withstand a pathological insult (1, 2, 17). There has been recent progress regarding the discovery of pathways and targets involved in neurodegenerative diseases and neuroprotection (Table 2).

**Table 2:** Pathways and targets in neurodegenerative diseases and neuroprotection.

**Pathway**	**Target**	**References**
Dendritic and microvascular dysfunction	Optic neuropathies	(16)
Lipid mediator signaling	Eye diseases	(68)
Apoptosis and cell death programs	Neuronal cells	(16, 69, 70)
Endoplasmic reticulum (ER) stress and the unfolded/misfolded protein response (UPR) pathways	Optic neuropathies and other neurodegenerative diseases	(71, 72)
Growth differentiating factors GDF-11 [bone morphogenetic protein (BMP)-11], and GDF-15 (BMP-15) pathways	RGC differentiation and development of the optic nerve and visual pathway	(73)
Inflammation	Eye and CNS diseases	(92–94)

These recent discoveries of the molecular mechanisms underlying optic neuropathies like glaucoma have not only provided new insights into the complex pathogenesis of such diseases, but have also afforded opportunities for the development of novel therapeutic strategies for sight preservation and/or vision restoration (74, 75).

##### Key considerations for targets in neuroprotection

Novel strategies to identify new targets in neuroprotection are needed (76). For example, newer testing methods should be leveraged, such as protein array analyses, single cell RNA sequencing, functional imaging (e.g., OCT), visually evoked potential (with electrodes), biofluid-based measures/biomarkers, reflex physiological measures, and automated high-throughput testing. In addition, new pathways and mediators should be explored, such a pro- and anti-inflammatory immune pathways and mediators, mitochondria/energy conservation, apoptosis/cell death, and unfolded/misfolded protein response pathways (77–81). Greater therapeutic efficacy may be achieved by addressing multiple pathways if a single molecule and/or pathway is unlikely to elicit complete therapeutic benefits (6–8, 65). A greater focus on master regulator targets that can influence multiple downstream pathways could result in therapeutic effects that extend beyond those associated with a single arm of a pathway (82). The use of pharmacodynamic biomarkers is essential to confirm target engagement (11, 83). These may be static (indicative of disease state) or dynamic (indicative of disease progression) (67). Understanding what the variability of a measurement is for a given effect size will determine biomarker utility. Pharmacodynamic biomarkers are needed to demonstrate a biological effect or determine the reason for trial failure (83, 84).

Viable therapeutic options for the effective treatment of optic neuropathies are currently lacking (74). However, there are several promising options under investigation, ranging from therapies aimed at overcoming metabolic defects and compensating for mitochondrial dysfunction, to gene therapies, to stem cell-based approaches (15). Several novel therapies have been investigated as potential areas to explore for further consideration in neuroprotection (6, 8, 13, 16, 74, 85).

##### Neurotrophic factors

Neurotrophic factors are a family of growth factors that regulate the survival, development, and differentiation of neurons, and many have been shown to promote RGC survival in experimental models of injury (2, 16). For example, a biological proteinaceous secretome from human amniotic progenitor cells containing growth factors and cytokines elicited anti-inflammatory and neuroprotective effects in a mouse model of multiple sclerosis (86). A proof-of-concept experiment involving intranasal delivery of an amnion-derived secretome in mice demonstrated prevention of visual function loss and RGC loss, as well as protection of visual function in the optic nerve (87). Neurotrophic factors are currently in human trials in glaucoma and other eye diseases, leveraging standard and exploratory biomarkers (88).

##### Inflammation

Historically, inflammation has been viewed solely as a neurodestructive event, with inflammatory cells, antibodies, and cytokines mediating loss of neurons and their connectivity. In some cases, inflammation may directly mediate the disease, following which neuronal loss follows (89). In other cases, specific categories of inflammation within the nervous system can either facilitate neuroprotection or mediate physiological or developmental neuronal mechanisms, such as complement-mediated synaptic pruning relevant to AD (90, 91). The general topic of inflammation in neurodegeneration has been extensively reviewed recently, given that it is likely to be a part of future neuroprotective strategies (92–94).

##### Electrical stimulation

Physiologic levels of electrical activity, whether provided through direct delivery of electrical current (95, 96) or through visual stimulation that the retina then converts into electrical stimulation (97), have also been identified as excellent candidates to promote neuronal survival and axon growth. The pathways by which electrical stimulation promote neuroprotection and regeneration include elevating specific second messenger pathways such as cyclic adenosine monophosphate (cAMP), enhancing neurotrophic factor responsiveness, and likely others (70, 96, 98).

Electrical stimulation has already demonstrated some levels of efficacy in human trials of patients with optic neuropathies, mainly using externally applied alternating current stimuli. For example, an average of 7–9% increase in visual field and a concomitant improvement in central visual acuity was reported in a large retrospective case series (99). Even more convincing, at least two masked, randomized controlled trials using high resolution perimetry and other visual field measures demonstrated significant improvement in scotomatous visual field areas and central acuity in the treated compared to sham control groups (100, 101). Equally encouraging have been the extended studies looking at concomitant enhancement of brain activity and circuity with electrical stimulation, e.g., using EEG (101–103). Measures such as alpha frequency on EEG may be both biomarkers of disease as well as endpoints for therapeutic efficacy (104). In most of these studies, patients were treated for a limited time, e.g., 2-week period; future work exploring whether longer courses of treatment could give larger, or more sustained efficacy will be important to explore.

##### Cell-based therapies

The replacement of diseased or degenerated cells by stem cell-derived RGCs could be a potential strategy to treat optic neuropathies. Stem cells are capable of differentiating into multiple cell types, thus having the potential to repair tissue and restore function after the development of lesions (2, 74). Experiments involving the transplantation of RGCs into uninjured rat retinas *in vivo* by intravitreal injection have demonstrated the promising potential of this approach as a cell replacement strategy (55). Transplantation was successful in terms of host retention, response to light stimulation, and axon growth along the optic nerve to appropriate target areas in the brain (55).

##### Key considerations for drugs/therapeutics in neuroprotection

Leveraging biomarkers is essential in the development of drugs and therapeutics in neuroprotection (11). A clear definition of the intended use of biomarkers could enable clarification of their role in therapy discovery and development (67). Identifying biomarkers that are translatable in parallel with the development of therapeutic candidates (bilateral biomarker development) is a sensible strategy. This approach would allow for characterization of drug-responsive signatures and provide greater confidence before advancing to human studies (11, 67, 105).

To expand opportunities to develop effective neuroprotective treatments, therapeutic candidates that target key pathways should be explored (13, 16, 68, 72), as well as addressing key criteria (65) (Table 3).

**Table 3:** Therapeutic targets for neuroprotection.

**Type of target**	**Target**
Pathway	Pathways that restore dendrite and capillary function to promote RGC survival
Pathway	Lipid mediators that promote inflammatory resolution and neuronal homeostasis
Pathway	Protein synthesis and folding pathways that prevent accumulation of misfolded proteins
Pathway	Stimulators of phagocytosis and/or autophagic mechanisms that can promote clearance of intracellular and extracellular debris including removal of damaged mitochondria, dead RGCs, injured or dying retinal interneurons, and malfunctioning components of the optic nerve / visual system
Pathway	Cellular energy conservation and cell death pathways that preserve neuronal cell survival and prevent the loss of synapses
Pathway	Factors and pathways that can protect existing cellular components of the RGC axons and optic nerve, and those that can restore damaged or missing elements, such as myelinating progenitor cells
Address criteria	Therapies associated with the resolution of chronic inflammation rather than inhibition of acute inflammation
Address criteria	Treatments that target existing disease and are not only preventive
Address criteria	Interventions that can be applied during the early disease stage
Address criteria	Treatments that promote overall retinal, thalamic and visual cortex neuronal and axonal health (132, 133)

Given the complexity of neurodegenerative diseases, multiple therapeutic agents may be needed to produce a clinical benefit, with each individual agent having a contributing effect (6–8, 65). Novel cell-based therapies should be explored, such as a mixture of cellular factors as a sort of combination therapy (e.g., secretome/exosomal vesicles) (86, 87). Optic nerve regeneration studies in humans are needed pending achievement of regulatory safety standards. Lastly, there is a need to review experiences from other disease states for lessons learned and templates to follow (106). The cancer field, for example, has a well-established path that outlines a timeline for therapy development (105).

#### Models

Research in the field of neuroprotection in glaucoma has been difficult, but new animal models and techniques will help bridge the gap between preclinical and clinical studies, and hopefully lead to clear beneficial outcomes (2, 66). There are currently several animal models of glaucoma used to study neuroprotection, including mice, rats, beagles, pigs, and non-human primates—each with advantages and disadvantages (4, 6, 8, 51, 107). Selecting appropriate models and testing them rigorously before proceeding to human trials would help to optimize dosing and delivery methods, and rule out species-specific effects that could make human trials less likely to succeed (4, 11, 51, 108, 109).

##### Key considerations for models in neuroprotection

For data reproducibility that gives greater confidence in preclinical findings, there is a need to use the same animal model across different laboratories (6, 7, 11). Conversely, the use of different animal models would allow for determination of whether specific biology translates across etiology using consistent methodology (11). For example, it would be ideal to observe a drug function across models which induce optic nerve inflammation by distinct mechanisms. As with biomarkers, there could be different uses for different models depending on the research question. There is a need to establish a set of guidelines for models when testing a drug or a biomarker, taking into consideration the different tools and models currently available (66). In terms of optic neuropathies, essential experimental models should be identified (e.g., axotomy, optic nerve crush, ocular hypertension induced by different means) (110). Animal age should also be considered when selecting a model and study design (11). For example, progression of glaucoma extends over a much longer period in humans compared with preclinical models (51).

There is precedent for standardization of translational research, based on experiences with epilepsy in the National Institute of Neurological Disorders and Stroke Epilepsy Therapy Screening Program (111, 112). The neurology community recognized decades ago a need for more therapies for seizures, and funded a facility where models were established and companies could provide candidate drugs for testing (111, 112). The program had well-characterized models and met requirements for moving forward with a particular drug (standardized for FDA approval). The program operates to this day, and dozens of drugs for epilepsy have resulted from it (111, 112).

#### Biomarkers

The availability of reliable biomarkers that track disease activity and correlate with clinical outcomes could enable homogenous recruitment of participants in future clinical studies, especially at the pre-symptomatic stage, potentially increasing opportunities to identify disease-modifying therapies (31, 33, 67, 83, 113). There are several promising new biomarkers in the field of neuroprotection, which have recently been comprehensively reviewed (57).

##### Detection of apoptosing retinal cells

DARC is a potential surrogate marker in glaucoma (17, 114). DARC has demonstrated promise in early phase trials. The technology enables the direct visualization of individual apoptosing retinal cells in both healthy subjects and patients with glaucoma, with good safety and tolerability (114, 115). In contrast to current diagnostic techniques, which allow observation of structural or functional damage that has already occurred, DARC enables direct observation of cellular apoptotic events in real-time. Those detected events could later contribute to the changes seen in OCT parameters and visual field tests (114). DARC technology provides an opportunity to monitor retinal disease activity at the *in vivo* level and longitudinally at the individual cell level. Thus, it may prove to be a useful adjunct to clinical endpoints when assessing treatment efficacy (114). DARC has the potential to be an early biomarker of glaucoma and may be predictive of future disease and its progression (114, 115).

##### Serum neurofilaments

In the last decade, serum neurofilaments (NFs) have emerged as a non-specific but sensitive biomarker of neurodegeneration across many neurological diseases (116). These neuron-specific structural components of motor axons are readily detected by conventional antibody-based immunoassays and are released into cerebrospinal fluid (CSF) and peripheral blood in a variety of pathological states, including ALS (58). NF biology appears to be generally consistent between animals and humans, and several studies on NFs in CSF have confirmed their diagnostic biomarker capabilities in distinguishing ALS from healthy controls or ALS mimic disorders (116). A recent prospective, multicenter, longitudinal study demonstrated that serum NFs may be considered a clinically validated prognostic biomarker for ALS with potential utility as a pharmacodynamic biomarker of treatment effect (117).

With recent technological advances in imaging, genomic, metabolomic, lipidomic, and proteomic techniques, potential new biomarkers may be identified (67). New biomarkers will require validation in large patient populations covering different stages of disease to determine their appropriate context of use in terms of being either diagnostic, predictive, prognostic, or indicative of therapeutic response (67, 113, 118).

##### Other promising biomarkers for the visual system

The eye is unusual in that the retina (which contains retinal neurons and the axons of the RGCs) can be directly imaged through the clear cornea and lens. The ability to image the retina has led to multiple recent or upcoming techniques that can be used as structural or functional biomarkers. Dynamic biomarkers of neuronal and retinal structure are straightforward to quantify, and include previously mentioned OCT of the RNFL, OCT of the combined ganglion cell layer and inner plexiform layer in the macula. The latter reflects loss of RGC somas, and not only axons as with the former, and is therefore more retinotopic.

Dynamic functional biomarkers of visual system disease can be used to study progressive diseases such as glaucoma and can go beyond standard visual acuity or visual field outcomes. These include multifocal visual evoked potentials, multifocal electroretinography, pattern electroretinography, OCT-angiography, laser speckle flowmetry, and retinal oximetry (119). The addition of eye tracking with microperimetry or in addition to standard visual field analysis are also effective biomarkers (120, 121).

Use of newer technologies, e.g., high resolution imaging using adaptive optics for structure and function, are likely to be available soon (122–124). The optoretinogram can be used to image photoreceptor function at high-resolution (125, 126), and should provide additional information about neurodegenerative processes in the eye. Imaging of intracellular calcium, reactive oxygen species, and other signaling biomarkers have been developed for animal imaging, but their use in humans depends on compounds that are safe for intravenous or intravitreal use (127, 128). An alternative is the use of narrow-band or multispectral imaging of retinal fluorescence, e.g., flavoprotein fluorescence as a marker of oxidative stress (129, 130).

##### Key considerations for biomarkers in neuroprotection

Key considerations for the development and use of biomarkers in neuroprotection are outlined below. Many of these considerations and themes have also been described elsewhere (51, 56, 66, 67, 83, 113, 131). Foremost are a biomarker's reliability, reproducibility, variability, characteristics, and the factors that influence these parameters. It is also important to differentiate between biomarkers that reflect disease activity and those that reflect disease severity, especially with respect to determining inclusion criteria for stage of disease or rates of progression.

##### Improving use of biomarkers

- Exploration of biomarkers in newly diagnosed patients (e.g., DARC).
- Use of similar biomarkers and testing methods across different models.
- Evaluation of regional and global biomarkers of structure and function.
- Use of biomarkers that are related to quality-of-life measures.
- Validation of early phase or pivotal trial endpoints and study design.
- Validation of any new proposed biomarker against a widely accepted point of comparison.

##### Differentiating biomarker purpose and use

- Consideration of a biomarker's context for use (e.g., predictive *vs*. prognostic).
- Biomarkers that correlate with functional outcomes are better suited as outcome measures (e.g., RNFL or RGC loss).
- Biomarkers that involve dynamic measures are better suited for patient stratification (e.g., DARC and laser speckle flowmetry).

##### Types of biomarkers that are needed

- Early biomarkers to better predict whether patients are progressing down a path of neurodegeneration (e.g., earlier detection of RGC damage or loss of visual field in glaucoma).
- Predictive biomarkers to identify patients most likely to benefit from therapy.
- Pharmacodynamic biomarkers should be prioritized to confirm target engagement.
- Early phase biomarkers that translate into a late-phase result to give more confidence in planning clinical trials.
- Biomarkers that are widely accepted by the community and eventually accepted as surrogates by regulatory authorities worldwide.
- Proof-of-concept biomarkers for eventual pivotal clinical trials.

##### Using biomarkers in clinical studies

- Integrate the use of biomarkers to improve the quality of translation; include a combination of functional and structural measurements for better correlation.
- Perform longitudinal studies and expand clinical databases.
- Perform a multicenter multimodal study to compare different biomarkers head-to-head.
- Mine existing multicenter data retrospectively and use to discover new biomarkers.

##### Using a clear methodology in biomarker studies

- Recognize the importance of statistical modeling in the biomarker development process.
- Utilize equal rigor for biomarker measurements and clinical assessments to minimize data variability and maintain consistency across early-phase and late-phase trials.
- Perform tests with a greater frequency to reduce noise.
- Ensure reproducibility in multiple centers on a worldwide basis.

##### Need for proof-of-concept biomarkers

- Acquire a better understanding of how well a biomarker translates from preclinical models to clinical disease in humans.
- Use other indicators of treatment efficacy (e.g., blood flow) and cross-validate them to gain further confidence in the results.
- Develop biomarkers that help define *in vivo* biology to enable differentiation between disease subtypes for diagnosis and prognosis.
- Improve collaborations/synergy between those research groups that focus on biomarkers and those that focus on disease mechanisms.
- Clinical studies should be given financial resources to collect specimens that enable biomarker development, which can be achieved by collaborations between academic institutions and pharmaceutical companies in neuro-ophthalmology trials.
- All studies should have a biomarker component to enable an accumulation of data on a longitudinal basis.
- Clinical trials should use more biomarkers to obtain better datasets that could inform future trial designs.
- Every neuroprotection trial should assess target engagement (PK/PD) for the drug being studied.

#### Clinical trials and the regulatory pathway

Clinical trials have the potential to significantly impact patient care, and thus should be designed to achieve this goal (48). With regard to pivotal phase 3 clinical trials, regulatory agencies will rarely approve a therapy if either of 2 large randomized masked clinical trials fails to show a predefined level of efficacy. If both trials fail to demonstrate efficacy, development of a therapy is often halted (47). In order to achieve the ultimate goal of introducing neuroprotective treatments in the clinic, a multidisciplinary approach involving basic scientists, clinicians, patients, regulatory specialists, and financial backers is needed to design research studies with the potential for rapid translation (41).

Improving translational research will require careful reflection, planning, literature searches, and assessment of the likelihood of success before committing to a large-scale trial (40). Experienced clinician-scientists in neurology and ophthalmology should be consulted and engaged in trial design, if possible, to overcome potential institutional bias. More comprehensive integration of evidence from various preclinical and clinical studies could facilitate the refinement of objectives and improve the translation of preclinical findings (40, 66).

##### Key considerations for clinical trials in neuroprotection

Key considerations for clinical trials in neuroprotection are outlined below. Many of these considerations and themes have also been described elsewhere (17, 51, 56, 65, 66, 83, 131).

##### Considerations at the patient level

- Patient selection based on genetics (i.e., Parkinson's disease).
- Reducing patient heterogeneity (e.g., identifying presymptomatic patients, age-matching).
- Use of an enriched population before the onset of significant disease can increase the ability to observe a therapeutic effect.
- Studying a more defined subgroup population rather than conducting large trials.
- Performing a large multicenter screening of prospective patients for larger trials.

##### Considerations at the trial design level

- Using novel trial designs (e.g., adaptive clinical trials that permit changes during the trials based on interim findings).
- Finding and developing appropriate objective measures; increasing the frequency of assessments (e.g., home testing), which has many advantages (e.g., shorter trial duration, earlier detection of events, reduced variability and greater power, cost effectiveness, and easier management).
- Need for proof-of-concept trials to better inform future pivotal trials; consideration for performing a series of smaller, shorter trials to test a variety of therapies and mechanisms rather than a few large trials.
- Every trial should integrate a biomarker study component to ensure lessons can be learned and knowledge leveraged for optimizing future trials.
- Using patient enrichment strategies to shorten duration or timeframe for study of neuroprotective agents.
- Using artificial intelligence to predict useful and approvable endpoints and to shorten clinical trial duration.

## Conclusions

There are many commonalities between the neurology and ophthalmology fields in terms of pathophysiology of neurodegeneration, therapy development, and translational challenges. Effective neuroprotection will likely involve the use of multiple therapies to produce significant and clinically meaningful outcomes. There are many lessons to be learned in terms of optimizing the use of biomarkers for both structure and function, their utility in early phase trials or eventually potential pivotal endpoints, and patient stratification. Conducting a large multimodal multi-biomarker patient study could enable comparison and measurement of biomarkers and help elucidate how emerging biomarkers correlate with current gold standard endpoints and thus provide supportive data in future trials. To improve the translational potential of emerging neuroprotective therapies, studies must accurately assess target engagement in order to test hypotheses and enable determination of why a trial succeeded or failed. Developing clinically effective therapies for optic neuropathies will require a collaborative approach focusing on different types of biomarkers, key biological pathways and upstream targets, and novel therapeutic strategies and modalities together with their optimal routes of administration to ensure target engagement.

## Author contributions

LL, NC, NS, and JG made substantial contributions to the design of the review. CP was involved in the drafting and editing of the text. All authors drafted and substantially revised the work, approved the submitted version, and have agreed both to be personally accountable for their contributions and to ensure that questions related to the accuracy or integrity of any part of the work, even ones in which the author was not personally involved, are appropriately investigated, resolved, and the resolution documented in the literature.

## Funding

LL has received support from Canada Research Chairs, Canadian Institutes of Health Research (PJT-162396), and the Congressionally Directed Medical Research Programs through the Vision Research Program Focused Translational Team Science Award (FTTSA) (W81XWH-18-VRP-FTTSA). JG has received support from the National Eye Institute (P30-EY026877, R01-EY032416), the Gilbert Family Foundation, and Research to Prevent Blindness, Inc. The meeting noted below was funded by Santen Inc. Santen was not involved in the study design, collection, analysis, interpretation of data, the writing of this article or the decision to submit it for publication.

## Conflict of interest

LL is a consultant to Annexon, Prilenia, Janssen, Roche, Perfuse, Genentech, UNITY, Eyevensys, and Santen; is a member of the Scientific Advisory Board for the Gilbert Family Foundation and the Steering Committee for the National Eye Institute Audacious Goal Initiative; and holds patents assigned to the Wisconsin Alumni Research Foundation. CP is an employee of TELUS Health. NC and NS are employees of Santen. JG is a consultant to Annexon, Equinox, Novartis, ONL Therapeutics, ReNetX, Roche, Santen, Skye, Thea, TwentyTwenty, Zeiss; has equity in Emmecell and Peripherex; and holds patents assigned to Stanford University.

## Publisher's note

All claims expressed in this article are solely those of the authors and do not necessarily represent those of their affiliated organizations, or those of the publisher, the editors and the reviewers. Any product that may be evaluated in this article, or claim that may be made by its manufacturer, is not guaranteed or endorsed by the publisher.

## Abbreviations

AD: Alzheimer's disease
ALS: Amyotrophic lateral sclerosis
BMP: Bone morphogenetic protein
CNS: Central nervous system
DARC: Detection of apoptosing retinal cells
ER: Endoplasmic reticulum
FDA: Food and Drug Administration
GDF: Growth differentiating factors
IOP: Intraocular pressure
LHON: Leber hereditary optic neuropathy
NF: Neurofilaments
NAION: Non arteritic anterior ischemic optic neuropathy
OAG: Open-angle glaucoma
OCT: Optical coherence tomography
PD: Parkinson's disease
RGC: Retinal ganglion cell
RNFL: Retinal nerve fiber layer
SAP: Standard automated perimetry
UPR: Unfolded/misfolded protein response.
